# The NOAEL Equivalent of Environmental Cadmium Exposure Associated with GFR Reduction and Chronic Kidney Disease

**DOI:** 10.3390/toxics10100614

**Published:** 2022-10-15

**Authors:** Soisungwan Satarug, Aleksandra Buha Đorđević, Supabhorn Yimthiang, David A. Vesey, Glenda C. Gobe

**Affiliations:** 1 Kidney Disease Research Collaborative, Translational Research Institute, Brisbane 4102, Australia; 2 Department of Toxicology “Akademik Danilo Soldatović”, University of Belgrade-Faculty of Pharmacy, 11000 Belgrade, Serbia; 3 Occupational Health and Safety, School of Public Health, Walailak University, Nakhon Si Thammarat 80160, Thailand; 4 Department of Nephrology, Princess Alexandra Hospital, Brisbane 4102, Australia; 5 School of Biomedical Sciences, The University of Queensland, Brisbane 4072, Australia; 6 NHMRC Centre of Research Excellence for CKD QLD, UQ Health Sciences, Royal Brisbane and Women’s Hospital, Brisbane 4029, Australia

**Keywords:** benchmark dose, BMDL, BMDU, cadmium, creatinine clearance, chronic kidney disease, eGFR, NOAEL, urine cadmium

## Abstract

Cadmium (Cd) is a highly toxic metal pollutant present in virtually all food types. Health guidance values were established to safeguard against excessive dietary Cd exposure. The derivation of such health guidance figures has been shifted from the no-observed-adverse-effect level (NOAEL) to the lower 95% confidence bound of the benchmark dose (BMD), termed BMDL. Here, we used the PROAST software to calculate the BMDL figures for Cd excretion (E_Cd_) associated with a reduction in the estimated glomerular filtration rate (eGFR), and an increased prevalence of chronic kidney disease (CKD), defined as eGFR ≤ 60 mL/min/1.73 m^2^. Data were from 1189 Thai subjects (493 males and 696 females) mean age of 43.2 years. The overall percentages of smokers, hypertension and CKD were 33.6%, 29.4% and 6.2%, respectively. The overall mean E_Cd_ normalized to the excretion of creatinine (E_cr_) as E_Cd_/E_cr_ was 0.64 µg/g creatinine. E_Cd_/E_cr_, age and body mass index (BMI) were independently associated with increased prevalence odds ratios (POR) for CKD. BMI figures ≥24 kg/m^2^ were associated with an increase in POR for CKD by 2.81-fold (*p* = 0.028). E_Cd_/E_cr_ values of 0.38–2.49 µg/g creatinine were associated with an increase in POR for CKD risk by 6.2-fold (*p* = 0.001). The NOAEL equivalent figures of E_Cd_/E_cr_ based on eGFR reduction in males, females and all subjects were 0.839, 0.849 and 0.828 µg/g creatinine, respectively. The BMDL/BMDU values of E_Cd_/E_cr_ associated with a 10% increase in CKD prevalence were 2.77/5.06 µg/g creatinine. These data indicate that Cd-induced eGFR reduction occurs at relatively low body burdens and that the population health risk associated with E_Cd_/E_cr_ of 2.77–5.06 µg/g creatinine was not negligible.

## 1. Introduction

Environmental exposure to cadmium (Cd) is inevitable for most people because the metal is present in almost all food types [1,2,3]. The realization in the 1940s that the condition referred to as “itai-itai” disease was due to the consumption of rice heavily contaminated with Cd brought into focus the real threat to health posed by this metal [4,5]. Itai-itai disease is the most severe form of human Cd poisoning, characterized by severe damage to the kidneys and bones, resulting in multiple bone fractures due to osteoporosis and osteomalacia [4,5]. The pathologic symptoms of the itai-itai disease have been replicated in Cd-treated cynomolgus monkeys [6].

To safeguard against excessive dietary Cd exposure, health guidance such as a tolerable intake level of Cd was established [7]. The Joint FAO/WHO Expert Committee on Food Additives and Contaminants (JECFA) considered the kidney to be the critical target of Cd toxicity [8]. By definition, the provisional tolerable weekly intake (PTWI) for a chemical with no known biological function is an estimate of the amount that can be ingested weekly over a lifetime without appreciable health risk. Subsequently, the PTWI for Cd was amended to a tolerable monthly intake (TMI) of 25 μg per kg body weight per month, equivalent to 0.83 μg per kg body weight per day [8]. This tolerable intake level for Cd was derived from a risk assessment model that assumed an increase in excretion of β_2_-microglobulin (β_2_M) (E_β2M_) above 300 μg/g creatinine as the point of departure (POD) [8]. However, we have shown that such an increase in E_β2M_ reflected tubular dysfunction and nephron loss, evident from a reduction in estimated glomerular filtration rate (eGFR) to 60 mL/min/1.73 m^2^ or below [9,10]. In effect, a tolerable intake level of Cd derived from the E_β2M_-based POD is not sufficiently low to be without an impact on human health. 

Current evidence suggests that sufficient tubular injury disables glomerular filtration and leads to nephron atrophy and a decrease in GFR [11,12,13]. Accordingly, we argue that a reduction in eGFR due to Cd nephropathy could serve as the POD from which health guidance values should be derived. Owing to some shortcomings of the no-observed-adverse-effect level (NOAEL), the benchmark dose (BMD) has been used as the POD [7,14,15,16]. The BMD is a dose level, derived from an estimated dose–response curve, associated with a specified change in response, termed benchmark response (BMR) which can be set at 1%, 5%, or 10% as required [14,15,16]. 

The present study had two major aims. The first aim was to characterize a reduction in eGFR and risk factors of chronic kidney disease (CKD) in a sufficiently large group of people with a wide range of environmental Cd exposure. The risk factors considered included age, body mass index (BMI), smoking, hypertension, and Cd exposure measured as excretion of Cd (E_Cd_). The second aim was to compute the lower 95% confidence bound of BMD (BMDL) and the BMD upper confidence limit (BMDU) of E_Cd_ associated with eGFR reduction and an increase in the prevalence of CKD. 

## 2. Materials and Methods

### 2.1. Participants

To represent a large group of subjects with a wide range of environmental Cd exposure levels suitable for the dose–response analysis and health risk calculation, we assembled archived data from 1189 persons who participated in large population-based studies undertaken in a Cd contamination area in the Mae Sot District, Tak Province (*n* = 537), and low exposure locations in Bangkok and Nakhon–Si–Thammarat Province (*n* = 652). The Institutional Ethical Committees of Chulalongkorn University, Chiang Mai University and the Mae Sot Hospital approved the study protocol for the Mae Sot and Bangkok groups. The Office of the Human Research Ethics Committee of Walailak University in Thailand approved the study protocol for the Nakhon Si Thammarat group [17,18]. 

All participants gave informed consent prior to participation. They had lived at their current addresses for at least 30 years. Exclusion criteria were pregnancy, breastfeeding, a history of metalwork, and a hospital record or physician’s diagnosis of advanced chronic disease. Because occupational exposure was an exclusion criterion, we presumed that all participants had acquired Cd from the environment. Diabetes was defined as fasting plasma glucose levels ≥ 126 mg/dL or a physician’s prescription of anti-diabetic medications. Hypertension was defined as systolic blood pressure ≥ 140 mmHg, diastolic blood pressure ≥ 90 mmHg, a physician’s diagnosis, or prescription of anti-hypertensive medications.

### 2.2. Collection and Analysis of Biological Specimens 

Simultaneous blood and urine sampling are required to normalize E_Cd_, to C_cr_. Accordingly, second-morning urine samples were collected after an overnight fast, and whole blood samples were obtained within 3 hours after the urine sampling. Aliquots of urine, whole blood and plasma were stored at −20 °C or −80 °C for later analysis. The assay for urine and plasma concentrations of creatinine ([cr]_u_ and [cr]_p_) was based on the Jaffe reaction. 

For the Bangkok group, urine concentration of Cd ([Cd]_u_) was determined by inductively-coupled plasma mass spectrometry (ICP/MS, Agilent 7500, Agilent Technologies, Santa Clara, CA, USA). Multi-element standards (EM Science, EM Industries, Inc., Newark, NJ, USA) were used to calibrate the Cd analyses. Quality assurance and control were conducted with simultaneous analyses of samples of the reference urine Lyphochek^®^ (Bio-Rad, Gladesville, New South Wales, Australia), which contained low- and high-range Cd levels. A coefficient of variation value of 2.5% was obtained for Cd in the reference urine. The low limit of detection (LOD) of urine Cd was 0.05 µg/L. The urine samples containing Cd below the LOD were assigned as the LOD divided by the square root of 2 [19].

For the Nakhon–Si–Thammarat group, [Cd]_u_ was determined with the GBC System 5000 Graphite Furnace Atomic Absorption Spectrophotometer (AAS) (GBC Scientific Equipment, Hampshire, IL, USA). Instrumental metal analysis was calibrated with multi-element standards (Merck KGaA, Darmstadt, Germany). Reference urine metal control levels 1, 2, and 3 (Lyphocheck, Bio-Rad, Hercules, CA, USA) were used for quality control, analytical accuracy, and precision assurance. The analytical accuracy of metal detection was checked by an external quality assessment every 3 years. The LOD of urine Cd was 0.1 µg/L. When [Cd]_u_ was below its detection limit, the Cd concentration assigned was the detection limit divided by the square root of 2 [19].

For the Mae Sot group, [Cd]_u_ was determined with AAS (Shimadzu Model AA-6300, Kyoto, Japan). Urine standard reference material No. 2670 (National Institute of Standards, Washington, DC, USA) was used for quality assurance and control purposes. The LOD of Cd quantitation, defined as 3 times the standard deviation of blank measurements was 0.06 µg/L. None of the urine samples from this group contained [Cd]_u_ below the detection limit.

### 2.3. Estimated Glomerular Filtration Rates (eGFR) 

The GFR is the product of nephron number and mean single nephron GFR, and in theory, the GFR is indicative of nephron function [20,21,22]. In practice, the GFR is estimated from established chronic kidney disease-epidemiology collaboration (CKD-EPI) equations and is reported as eGFR [21]. 

Male eGFR = 141 × [plasma creatinine/0.9]^Y^ × 0.993^age^, where Y = −0.411 if [cr]_p_ ≤ 0.9 mg/dL, Y = −1.209 if [cr]_p_ > 0.9 mg/dL. Female eGFR = 144 × [plasma creatinine/0.7]^Y^ × 0.993^age^, where Y = −0.329 if [cr]_p_ ≤ 0.7 mg/dL, Y = −1.209 if [cr]_p_ > 0.7 mg/dL. For dichotomous comparisons, CKD was defined as eGFR ≤ 60 mL/min/1.73 m^2^. CKD stages 1, 2, 3a, 3b, 4, and 5 corresponded to eGFR of 90–119, 60–89, 45–59, 30–44, 15–29, and <15 mL/min/1.73 m^2^, respectively. 

### 2.4. Normalization of E_Cd_ to E_cr_ and C_cr_

E_x_ was normalized to E_cr_ as [x]_u_/[cr]_u_, where x = Cd; [x]_u_ = urine concentration of x (mass/volume); and [cr]_u_ = urine creatinine concentration (mg/dL). The ratio [x]_u_/[cr]_u_ was expressed in μg/g of creatinine. 

E_x_ was normalized to C_cr_ as E_x_/C_cr_ = [x]_u_[cr]_p_/[cr]_u_, where x = Cd; [x]_u_ = urine concentration of x (mass/volume); [cr]_p_ = plasma creatinine concentration (mg/dL); and [cr]_u_ = urine creatinine concentration (mg/dL). E_x_/C_cr_ was expressed as the excretion of x per volume of filtrate [23]. 

### 2.5. Benchmark Dose Computation and Benchmark Dose–Response (BMR) Setting 

We used the web-based PROAST software version 70.1 (https://proastweb.rivm.nl accessed on 13 October 2022) to compute the BMD figures for E_Cd_/E_cr_ and E_Cd_/C_cr_ associated with glomerular dysfunction. A specific effect size termed the benchmark response (BMR) was set at 5% for a continuous eGFR reduction endpoint and at 10% for a quantal endpoint where eGFR ≤ 60 mL/min/1.73 m^2^. For a continuous endpoint, BMD values were computed from fitting datasets to four dose–response models, including inverse exponential, natural logarithmic, exponential, and Hill models. For a quantal endpoint, BMD values were calculated from fitting datasets to seven dose–response models that included two-stage, logarithmic logistic, Weibull, logarithmic probability, gamma, exponential and Hill models. The BMD 95% confidence intervals of E_Cd_/E_cr_ and E_Cd_/C_cr_ were from model averaging using bootstrap with 200 repeats.

The BMDL and BMDU corresponded to the lower bound and upper bound of the 95% confidence interval (CI) of BMD. The wider the BMDL-BMDU difference, the higher the statistical uncertainty in the dataset [23,24,25,26]. BMDL/BMDU figures of E_Cd_ for the glomerular endpoint were calculated for males, females and all subjects. 

### 2.6. Statistical Analysis

Data were analyzed with IBM SPSS Statistics 21 (IBM Inc., New York, NY, USA). The one-sample Kolmogorov–Smirnov test was used to identify departures of continuous variables from a normal distribution, and a logarithmic transformation was applied to variables that showed rightward skewing before they were subjected to parametric statistical analysis. The Mann–Whitney U-test was used to compare mean differences between the two groups. The Chi-square test was used to determine differences in percentage and prevalence data. The multivariable logistic regression analysis was used to determine the Prevalence Odds Ratio (POR) for CKD in relation to six independent variables; age, BMI, gender, smoking, hypertension and Cd exposure measures as E_Cd_. We employed two models in each logistic regression analysis: model 1 incorporated log_2_(E_Cd_/Ecr) or three E_Cd_/E_cr_ groups; model 2 incorporated log_2_(E_Cd_/C_cr_) or three E_Cd_/C_cr_ groups. All other independent variables in models 1 and 2 were identical. For all tests, *p*-values ≤ 0.05 for two-tailed tests were assumed to indicate statistical significance.

## 3. Results

### 3.1. Characterization of Cadmium Exposure by Sex and Smoking

Table 1 provides demographic data of participants (493 males and 696 females) stratified by sex and smoking status. 

The overall mean age of participants was 43.2 years, and the overall percentages of current smokers plus those who had stopped smoking for less than 10 years, hypertension and low eGFR were 33.6%, 29.4% and 6.2%, respectively. The overall mean [Cd]_u_ and mean E_Cd_/E_cr_ were 0.94 µg/L and 0.64 µg/g creatinine, while the overall mean E_Cd_/C_cr_ × 100 was 1.02 µg/L filtrate.

Smoking was higher among males (57.4%) than females (16.4%). In both sexes, % of smokers and non-smokers with hypertension did not differ. However, % of low eGFR among smokers was 3.7- and 3.8-fold higher than non-smokers in female and male groups, respectively. For the female group only, the mean BMI was 6 % lower in smokers than non-smokers (*p* = 0.004). 

For the male group, the mean [Cd]_u_ in smokers was 5.4-fold higher than nonsmokers (1.73 vs. 0.32 µg/L, *p* < 0.001). Mean E_Cd_/E_cr_ and mean E_Cd_/C_cr_ in smokers were 2.9- and 4.1-fold higher than in nonsmokers, respectively.

For the female group, the mean [Cd]_u_ in smokers was 6.4-fold higher than nonsmokers (4.84 vs. 0.75 µg/L, *p* < 0.001). Mean E_Cd_/E_cr_ and mean E_Cd_/C_cr_ in smokers were 3.2-and 6-fold higher than in nonsmokers, respectively.

### 3.2. Characterization of CKD Risk factors

Table 2 provides the results of a logistic regression analysis where E_Cd_/E_cr_ and E_Cd_/C_cr_ were continuous variables, while age and BMI were categorical variables.

An independent effect on the POR for CKD was observed for E_Cd_/E_cr_, BMI and age (Table 2). Sex, smoking and hypertension were not associated with the POR for CKD. Doubling of E_Cd_/E_cr_ was associated with an increase in POR for CKD by 1.47-fold (*p* < 0.001). BMI figures ≥ 24 kg/m^2^ were associated with 2.81-fold increase in POR for CKD (*p* = 0.028). Compared with those aged 16–45 years, the POR values for CKD were 14-, 28- and 141-fold higher in those aged 46–55, 56–65, and 66–87 years, respectively.

In an equivalent analysis of the C_cr_-normalized datasets, E_Cd_/C_cr_, BMI and age were independently associated with increased POR for CKD. Sex, smoking and hypertension were not associated with the POR for CKD. Doubling of E_Cd_/C_cr_ was associated with an increase in POR for CKD by 1.96-fold (*p* < 0.001). BMI figures ≥ 24 kg/m^2^ were associated with a 3.12-fold increase in POR for CKD (*p* = 0.022). Compared with those aged 16–45 years, the POR values for CKD were 10-, 35- and 199-fold higher in those aged 46–55, 56–65, and 66–87 years, respectively. 

### 3.3. Cadmium Excretion in Relation to the Risk of CKD 

Table 3 provides the results of a logistic regression analysis where age and BMI were continuous variables, while E_Cd_/E_cr_ was a categorical variable in model 1, and E_Cd_/C_cr_ was categorical in model 2.

Age and BMI were independently associated with increased POR for CKD in both models 1 and 2. Compared with E_Cd_/E_cr_ ≤ 0.37 µg/g creatinine (model 1), the POR for CKD was increased by 6.2- and 10.6-fold in those with E_Cd_/E_cr_ values of 0.38–2.49 and ≥2.5 µg/g creatinine, respectively. Compared with E_Cd_/C_cr_ ≤ 9.9 ng/L filtrates (model 2), the POR for CKD was increased by 4.4- and 20.8-fold in those with E_Cd_/C_cr_ values of 10–49.9 and ≥50 ng/L filtrate, respectively.

### 3.4. BMDL/BMDU Figures of E_Cd_ Associated with Reduced Glomerular Function 

#### 3.4.1. E_cr_-Normalized Dataset

**Figure 1:**
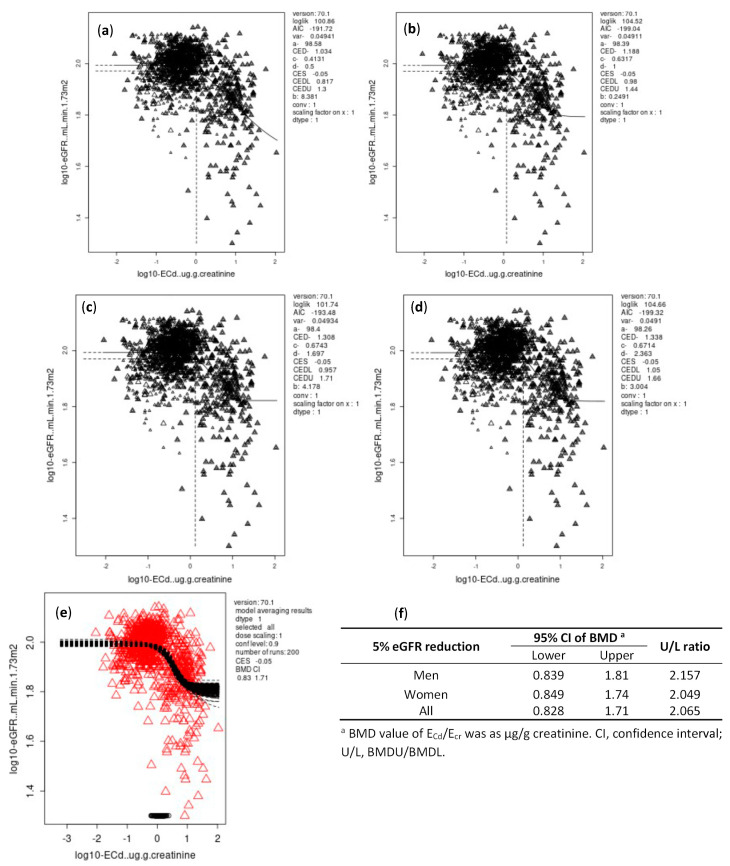
BMD estimates of E_Cd_/E_cr_ from eGFR reduction endpoint with BMR at 5%. E_Cd_/E_cr_ and eGFR data were fitted to an inverse exponential model (**a**), a natural logarithmic model (**b**), an exponential model (**c**), and Hill model (**d**). Bootstrap curves were based on model averaging of E_Cd_/E_cr_ BMD estimates for all subjects (**e**). Outputs of all fitted models as BMDL and BMDU estimates of E_Cd_/E_cr_ associated with a 5 % reduction in eGFR (**f**).

**Figure 2:**
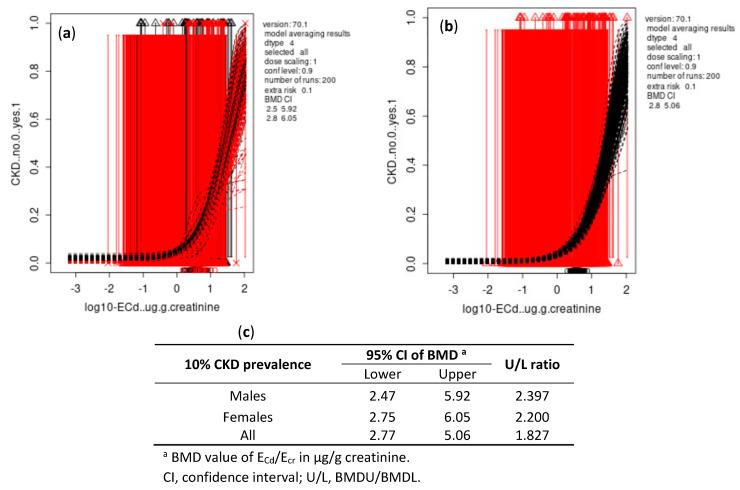
BMD estimates of E_Cd_/E_cr_ from quantal eGFR endpoint with BMR at 10%. Bootstrap curves were based on model averaging 95% confidence intervals of BMD of E_Cd_/E_cr_ in males and females (**a**) and in all subjects (**b**). Outputs of all fitted models as BMDL and BMDU estimates of E_Cd_/E_cr_ associated with a 10% increase in prevalence of CKD (**c**).

As data in Figure 1 and Figure 2 indicate, the differences between BMDL and BMDU figures of E_Cd_/E_cr_ were small for both continuous and quantal endpoints. The BMDL-BMDU figures of E_Cd_/E_cr_ calculated from Cd-dose and eGFR response models were higher in females than males.

For all subjects, the BMDL/BMDU of E_Cd_ /E_cr_ for continuous and quantal endpoints were 0.828/1.71 and 2.77/5.06 µg/g creatinine, respectively.

#### 3.4.2. C_cr_-Normalized Dataset 

**Figure 3:**
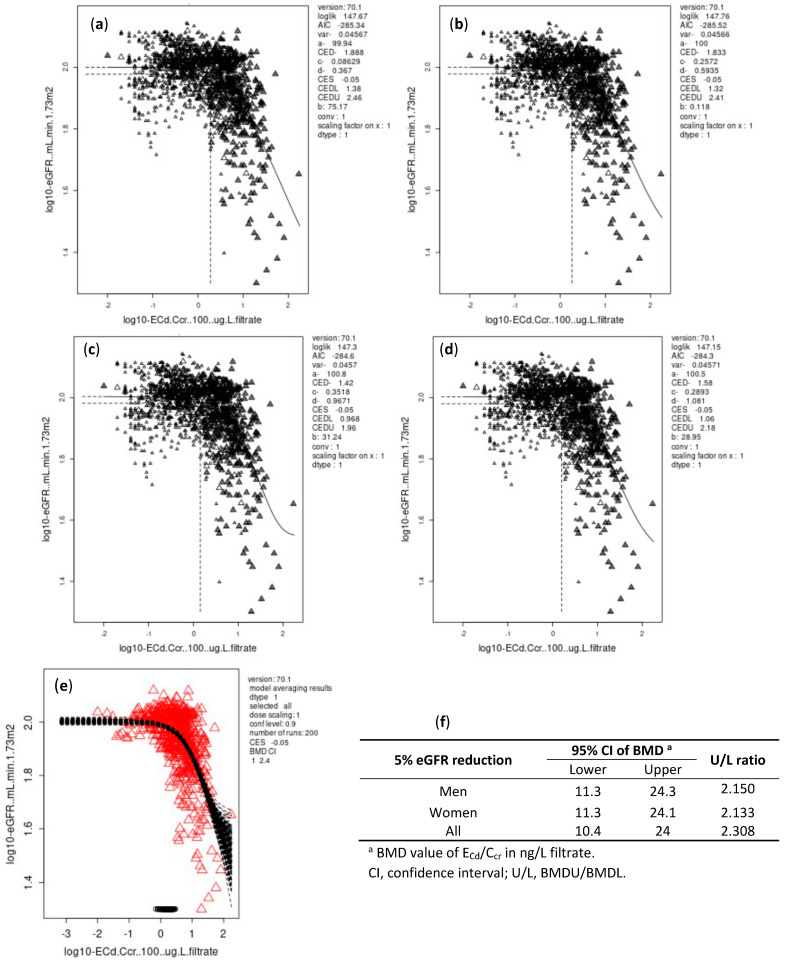
BMD estimates of E_Cd_/C_cr_ from eGFR reduction endpoint with BMR at 5%. E_Cd_/C_cr_ and eGFR data were fitted to an inverse exponential model (**a**), a natural logarithmic model (**b**), an exponential model (**c**), and Hill model (**d**). Bootstrap curves were based on model averaging of E_Cd_/C_cr_ BMD estimates for all subjects (**e**). Outputs of all fitted models as BMDL and BMDU estimates of E_Cd_/C_cr_ associated with a 5% reduction in eGFR (**f**).

**Figure 4:**
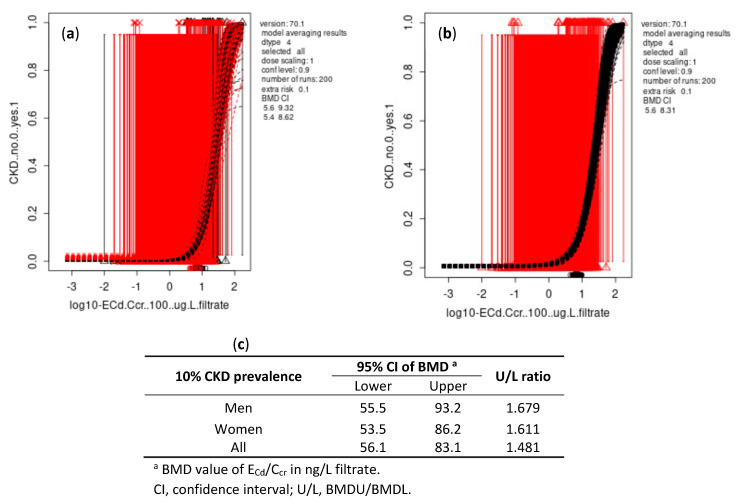
BMD estimates of E_Cd_/C_cr_ from quantal eGFR endpoint with BMR at 10%. Bootstrap curves were based on model averaging 95% confidence intervals of BMD of E_Cd_/C_cr_ in males and females (**a**) and in all subjects (**b**). Outputs of all fitted models as BMDL and BMDU estimates of E_Cd_/E_cr_ associated with a 10% increase in the prevalence of CKD (**c**).

As data in Figure 3 and Figure 4 indicate, the differences between BMDL and BMDU figures of E_Cd_/C_cr_ were small for both continuous and quantal endpoints. The BMDL-BMDU figures of E_Cd_/E_cr_ calculated by Cd-dose and eGFR response models in males and females were nearly identical. 

For all subjects, the BMDL/BMDU of E_Cd_ /C_cr_ for continuous and quantal endpoints were 10.4/24 and 56.1/83.1 ng/L filtrate, respectively.

## 4. Discussion

In a dose–response analysis of a large dataset from apparently healthy participants (mean age 48.3 years), older age and higher BMI were independently associated with higher risks of CKD, based on the low eGFR criterion (Table 2). These findings are consistent with the literature reports of age, overweight and obesity as common CKD risk factors [27,28,29,30]. In addition to these two risk factors, we have found the measure of long-term exposure to Cd (E_Cd_/E_cr_) to be another independent risk factor of CKD (Table 3). An association between low environmental Cd exposure and a decrease in eGFR to levels commensurate with CKD has been observed in population-based studies in the U.S. [31,32,33,34], Taiwan [35] and Korea [36,37,38].

In this study, the risk of CKD was increased by 6.2- and 10.6-fold, when E_Cd_/E_cr_ ≤ 0.37 µg/g creatinine rose to 0.38–2.49 and ≥ 2.5 µg/g creatinine, respectively. These Cd-dose dependent increases in the risk of CKD were strengthened by the results obtained from the C_cr_-normalized dataset where the risk of CKD was increased by 4.4- and 20.8-fold, comparing E_Cd_/C_cr_ ≤ 9.9 ng/L filtrates with E_Cd_/C_cr_ of 10–49.9 and ≥50 ng/L filtrate, respectively. This confirmation is noteworthy because normalizing E_Cd_ to E_cr_ can cause a wide dispersion of dataset due to the interindividual differences in E_cr_ such as muscle mass which is unrelated to neither Cd exposure nor nephron function [11,12]. 

Because of such increased variance in datasets introduced by E_cr_-normalization, the effect of chronic exposure to low-dose Cd on eGFR was not realized. For example, a systematic review and meta-analysis of pooled data from 28 studies reported that the risk of proteinuria was increased by 1.35-fold when comparing the highest vs. lowest category of Cd dose metrics, but an increase in the risk of low eGFR was statistically insignificant (*p* = 0.10) [39]. An erroneous conclusion that chronic Cd exposure was not associated with a progressive eGFR reduction was also made in another systematic review [40].

A significant relationship was seen between E_Cd_ and a decrease in eGFR with adjustment for covariates (Table 3). We subsequently applied the BMD method to our E_cr_- and C_cr_-normalized datasets to identify E_Cd_/E_cr_ and E_Cd_/C_cr_ values below which an adverse effect of Cd on eGFR can be discerned. The BMDL/BMDU figures of E_Cd_/E_cr_, estimated from the eGFR reduction endpoint were 0.839/1.81, 0.849/1.74 and 0.828/1.71 µg/g creatinine in males, females and all subjects, respectively (Figure 1). The corresponding BMDL/BMDU figures of E_Cd_/C_cr_ were 11.3/24.3, 11.3/24.1 and 10.4/24 ng/L filtrate in males, females and all subjects, respectively (Figure 3). 

The BMD values of Cd exposure levels calculated from toxic tubular cell injury and reduced tubular reabsorption of the filtered protein β_2_M can be found in numerous studies [41,42]. In contrast, a report of BMDL/BMDU of Cd exposure levels associated with eGFR reduction could only be found in a study of 790 Swedish women, aged 53–64 years, where the reported BMDL values for the glomerular endpoint were 0.7–1.2 μg/g creatinine [43]. These BMD values were slightly lower than those calculated for females in the present study (0.849/1.74 μg/g creatinine). The differences may be attributable to lower E_cr_ in Thai women than in Swedish women. Nevertheless, all these BMD values were lower than E_Cd_/E_cr_ of 5.24 µg/g creatinine, which suggested to be a threshold level for the nephrotoxicity of Cd when E_β2M_/E_cr_ > 300 was used as the POD [8]. 

In our quantal eGFR endpoint analysis (Figure 2), the BMDL/BMDU values of E_Cd_ associated with a 10% increase in CKD prevalence were 2.77/5.06 µg/g creatinine (56.1/83.1 ng/L filtrate). These data suggested that population CKD prevalence was likely to be smaller than 10% at E_Cd_/E_cr_ < 2.77 µg/g creatinine (<56.1 ng/L filtrates). Thus, the population health risk associated with E_Cd_/E_cr_ < 2.77 µg/g creatinine could not be discerned. The impact of Cd exposure on GFR has long been underestimated due to the common practice of normalizing E_Cd_ to E_cr_. The comparability of guidelines between populations could be improved by the universal acceptance of a consistent normalization of E_Cd_ to Ccr that eliminates the effect of muscle mass on E_cr_, thereby giving a more accurate assessment of the severity of Cd nephropathy [10].

A tolerable intake level of 0.28 μg/kg body weight per day was derived in a risk calculation using pooled data from Chinese population studies [44]. This consumption level, equivalent to 16.8 µg/day for a 60 kg person, was derived from an E_β2M_/E_cr_ endpoint where the BMDL value of E_Cd_/E_cr_ for such an endpoint was 3.07 μg/g creatinine. This BMDL estimate was 3.7-fold higher than the BMDL of 0.828 µg/g creatinine derived in the present study. In another Chinese population study, dietary Cd intake estimates at 23.2, 29.6, and 36.9 μg/d were associated with 1.73-, 2.93- and 4.05-fold increments in the prevalence of CKD, compared with the 16.7 μg/d intake level [45]. A diet high in rice, pork, and vegetables was associated with a 4.56-fold increase in the prevalence of CKD [45].

The European Food Safety Authority (EFSA) also used the β_2_M endpoint. However, the EFSA included an uncertainty factor (safety margin), and an intake of 0.36 μg/kg body weight per day for 50 years as an acceptable Cd ingestion level or a reference dose (RfD) [46]. The EFSA designated E_Cd_/E_cr_ of 1 μg/g creatinine as the toxicity threshold level for an adverse effect on kidneys. This Cd excretion of 1 μg/g creatinine is 17 % higher than our NOAEL equivalent of Cd excretion of 0.828 µg/g creatinine.

The Cd toxicity threshold level, RfD and an acceptable consumption level derived from the β_2_M excretion above ≥300 µg/ g creatinine do not appear to be without an appreciable health risk. In theory, health-risk assessment should be based on the most sensitive endpoint with consideration given to subpopulations with increased susceptibility to Cd toxicity such as children. 

In the present study, the body burden of Cd, measured as E_Cd_/E_cr_, was increased by 3-fold in men and women who smoked cigarettes (Table 1). These results are expected, given that the tobacco plant accumulates high levels of Cd in its leaves, and the volatile metallic Cd and oxide (CdO) generated from cigarette burning are more bioavailable than Cd that enters the body through the gut [47,48].

The diet is the main Cd exposure source for non-smoking and non-occupationally-exposed populations. In a temporal trend analysis of environmental Cd exposure in the U.S., the mean urinary Cd fell by 29% in men (0.58 vs. 0.41 μg/g creatinine, *p* < 0.001) over 18 years (NHANES 1988–2006), but not in women (0.71 vs. 0.63 μg/g creatinine, *p* = 0.66) [49]. Such a reduction in Cd exposure among men was attributable to a decrease in smoking prevalence [50]. In contrast, total diet studies in Australia [51], France [52], Spain [53] and the Netherlands [2] reported that dietary Cd exposure levels among young children exceeded the current health guidance values. These data are concerning for the reasons below.

CKD is a progressive syndrome with high morbidity and mortality and affects 8% to 16% of the world’s population [27,28,29,30]. An upward trend of its incidence continues, while an adverse effect of Cd on eGFR and the risk of CKD have increasingly been reported. Higher Cd excretion was associated with lower eGFR in studies from Guatemala [54] and Myanmar [55]. The effect of Cd exposure on eGFR observed in children is particularly concerning. In a prospective cohort study of Bangladeshi preschool children, an inverse relationship between urinary Cd excretion and kidney volume was seen in children at 5 years of age. This was in addition to a decrease in eGFR [56]. Urinary Cd levels were inversely associated with eGFR, especially in girls. In another prospective cohort study of Mexican children, the reported mean for Cd intake at the baseline was 4.4 µg/d, which rose to 8.1 µg/d after nine years, when such Cd intake levels showed a marginally inverse association with eGFR [57]. 

## 5. Conclusions

Environmental exposure to Cd, old age, and elevated BMI are independent risk factors for reduced eGFR. For the first time, the BMDL/BMDU figures of Cd excretion levels associated with a decrease in eGFR have been computed for men and women. The narrow BMDL-BMDU differences indicate the high degree of statistical certainty in these derived NOAEL equivalent figures. The BMDL/BMDU estimates of the Cd excretion associated with a decrease in eGFR in all subjects are 0.828/1.71 µg/g creatinine. The BMDL/BMDU estimates of Cd excretion associated with a 10% increase in the prevalence of CKD are 2.77/5.06 µg/g creatinine. These NOAEL equivalents indicate a decrease in eGFR due to Cd nephropathy occurs at the body burdens lower than those associated with Cd excretion of 5.24 µg/g creatinine and an increase in β_2_M excretion above 300 µg/g creatinine. The established nephrotoxicity threshold level for Cd is outdated and is not protective of human health. Human health risk assessment should be based on current scientific research data.

## Figures and Tables

**Table 1:** Characteristics of participants stratified by sex and smoking status.

Parameters	All subjects *n* 1189 (33.6% Smokers)	Males, *n* 493 (57.4% Smokers)		Females, *n* 696 (16.8% Smokers)	
		Nonsmokers *n* 210	Smokers *n* 283	Nonsmokers *n* 579	Smokers *n* 117
Age, years	43.2 ± 14.0	35.9 ± 13.0	45.0 ± 14.8 ***	42.6 ± 12.9	54.2 ± 10.1 ^###^
Hypertension (%)	29.4	26.5	27.2	30.8	33.3
BMI, kg/m^2^	23.0 ± 3.9	22.4 ± 3.0	22.3 ± 3.4	23.8 ± 4.0	22.4 ± 4.6 ^#^
BMI groups (%)					
12–18	10.5	9.2	12.2 *	7.4	21.4
19–23	47.1	56.9	57.2 **	41.6	36.8 ^###^
≥24	42.4	34.0	30.6	51.0	41.9 ^###^
eGFR ^a^, mL/min/1.73 m^2^	93.7 ± 20	96.6 ± 17.6	91.9 ± 22.1	95.9 ± 19.3	81.8 ± 22.0 ^###^
eGFR ≤ 60 mL/min/1.73 m^2^ (%)	6.2	2.4	8.8 **	4.3	16.2 ^###^
eGFR, mL/min/1.73 m^2^ (%) ^b^					
>120	7.8	9.0	5.7	9.7	1.7 ^###^
90–120	53.8	61.0	56.5	54.1	33.3 ^###^
60–89	32.8	27.6	29.7 *	32.8	49.6 ^###^
30–59	5.0	1.9	7.1 **	3.3	13.7
15–29	0.6	0.5	1.1	0.2	1.7
Plasma creatinine, mg/dL	0.88 ± 0.24	1.00 ± 0.21	1.00 ± 0.27	0.76 ± 0.16	0.82 ± 0.27 ^##^
Urine creatinine, mg/dL	104.4 ± 73.5	81.1 ± 78	107.0 ± 75.6 ***	67.9 ± 68.9	79.8 ± 64.8
Urine Cd, µg/L	0.94 ± 9.69	0.32 ± 5.96	1.73 ± 15.9 ***	0.75 ± 6.46	4.84 ± 6.38 ^###^
Normalized to E_cr_ as E_x_/E_cr_^c^					
E_Cd_/E_cr_, µg/g creatinine	0.64 ± 6.12	0.32 ± 3.29	0.94 ± 8.85 ***	0.57 ± 4.62	1.83 ± 7.27 ^###^
Normalized to C_cr_ as E_x_/C_cr_ ^d^					
E_Cd_/C_cr_ × 100, µg/L filtrate	1.02 ± 8.19	0.39 ± 4.75	1.61 ± 12.61 ***	0.83 ± 5.27	5.00 ± 9.36 ^###^

*n*, number of subjects; BMI, body mass index; eGFR, estimated glomerular filtration rate; E_x_, excretion of x; cr, creatinine; C_cr_, clearance of creatinine. ^a^ eGFR determined with Chronic Kidney Disease Epidemiology Collaboration (CKD–EPI) equations [20]; ^b^ eGFR of 90–119, 60–89, 45–59, 30–44, 15–29, and <15 mL/min/1.73 m^2^ corresponded to CKD stages 1, 2, 3a, 3b, 4, and 5, respectively. ^c^ E_x_/E_cr_ = [x]_u_/[cr]_u_; ^d^ E_x_/C_cr_ = [x]_u_[cr]_p_/[cr]_u_, where x = Cd [23]. Data for age, eGFR and BMI are arithmetic means ± standard deviation (SD). Data for all other continuous variables are geometric means ± SD. Data for BMI are from 951 subjects; data for hypertension are from 917 subjects; data for all other variables are from 1189 subjects. For each test, *p* ≤ 0.05 identifies statistical significance, determined by Chi-Square test and Mann–Whitney U test for % differences and mean differences, respectively. Compared with non-smoking males * *p* = 0.029–0.042, ** *p* = 0.001–0.006, *** *p* ≤ 0.001. Compared with non-smoking females, ^#^ *p* = 0.004, ^##^ *p* = 0.001, ^###^ *p* ≤ 0.001.

**Table 2:** Increment in risk of chronic kidney disease in relation to age, BMI and cadmium exposure.

Independent Variables/ Factors	Number of Subjects	^a^ CKD				
		β Coefficients	POR	95% CI		*p*
		(SE)		Lower	Upper	
*Model 1*						
Log_2_[(E_Cd_/E_cr_) × 10^3^], µg/g creatinine	917	0.385 (0.072)	1.470	1.276	1.692	<0.001
Hypertension	276	0.490 (0.312)	1.632	0.885	3.008	0.117
Gender (female)	562	0.028 (0.340)	1.029	0.528	2.002	0.934
Smoking	335	0.209 (0.337)	1.232	0.637	2.383	0.536
BMI, kg/m^2^						
12–18	99	Referent				
19–23	431	0.057 (0.426)	1.058	0.459	2.439	0.894
≥24	387	1.033 (0/470)	2.810	1.118	7.064	0.028
Age, years						
16–45	392	Referent				
46–55	348	2.655 (1.036)	14.23	1.867	108.4	0.010
56–65	100	3.340 (1.059)	28.21	3.538	224.9	0.002
66–87	77	4.950 (1.055)	141.2	17.87	1116	<0.001
*Model 2*						
Log_2_[(E_Cd_/C_cr_) × 10^5^], µg/L filtrate	917	0.674 (0.107)	1.962	1.589	2.422	<0.001
Hypertension	276	0.551 (0.326)	1.735	0.916	3.287	0.091
Gender (female)	562	−0.174 (0.366)	0.840	0.410	1.719	0.633
Smoking	335	−0.058 (0.351)	0.944	0.474	1.879	0.869
BMI, kg/m^2^						
12–18	99	Referent				
19–23	431	0.103 (0.457)	1.109	0.452	2.717	0.822
≥24	387	1.147 (0.500)	3.150	1.181	8.400	0.022
Age, years						
16–45	392	Referent				
46–55	348	2.298 (1.036)	9.951	1.305	75.88	0.027
56–65	100	3.543 (1.062)	34.57	4.312	277.2	0.001
66–87	77	5.292 (1.066)	198.6	24.59	1605	<0.001

POR, Prevalence Odds Ratio; S.E., standard error of mean; CI, confidence interval. ^a^ CKD was defined as estimated glomerular filtration rate (eGFR) ≤ 60 mL/min/1.73 m^2^. Coding; female = 1, male = 2, hypertensive = 1, normotensive = 2, smoker = 1, non-smoker = 2. Data were generated from logistic regression analyses relating POR for CKD to six independent variables, listed in the first column. For all tests, *p*-values < 0.05 indicate statistical significance. Log_2_[(E_Cd_/E_cr_) × 10^3^] was incorporated into model 1; log_2_[(E_Cd_/C_cr_) × 10^5^] was incorporated into model 2. Other independent variables in models 1 and 2 were identical. β coefficients indicate an effect size of each independent variable on POR for CKD.

**Table 3:** Dose–response relationship between cadmium excretion and the risk of chronic kidney disease.

Independent Variables/ Factors	Number of Subjects	^a^ CKD				
		β Coefficients	POR	95% CI		*p*
		(SE)		Lower	Upper	
*Model 1*						
Age, years	917	0.126 (0.016)	1.135	1.100	1.170	<0.001
BMI, kg/m^2^	917	0.082 (0.038)	1.086	1.009	1.169	0.028
Gender (female)	562	0.124 (0.337)	1.132	0.585	2.190	0.713
Hypertension	276	0.304 (0.310)	1.355	0.738	2.486	0.327
Smoking	335	0.173 (0.345)	1.189	0.605	2.338	0.615
E_Cd_/E_cr_, µg/g creatinine						
≤0.37	358	Referent				
0.38–2.49	333	1.819 (0.565)	6.164	2.035	18.67	0.001
≥2.5	226	2.362 (0.557)	10.61	3.562	31.60	<0.001
*Model 2*						
Age, years	917	0.141 (0.016)	1.152	1.116	1.189	<0.001
BMI, kg/m2	917	0.099 (0.039)	1.104	1.023	1.191	0.011
Gender (female)	562	0.191 (0.356)	1.211	.602	2.434	0.591
Hypertension	276	0.240 (0.314)	1.271	0.687	2.353	0.445
Smoking	335	−0.033 (0.359)	0.968	0.479	1.956	0.927
E_Cd_/C_cr_, ng/L filtrate						
≤9.9	346	Referent				
10–49.9	326	1.470 (0.642)	4.350	1.237	15.30	0.022
≥50	245	3.036 (0.637)	20.82	5.979	72.52	<0.001

POR, Prevalence Odds Ratio; S.E., standard error of mean; CI, confidence interval. ^a^ CKD was defined as estimated glomerular filtration rate (eGFR) ≤ 60 mL/min/1.73 m^2^. Coding; female = 1, male = 2, hypertensive = 1, normotensive = 2, smoker = 1, non-smoker = 2. Data were generated from logistic regression analyses relating POR for CKD to six independent variables listed in the first column. For all tests, *p*-values < 0.05 indicate statistical significance. Three E_Cd_/Ecr categories were incorporated into model 1; three E_Cd_/C_cr_ × 100 categories were incorporated into model 2. Other independent variables in models 1 and 2 were identical. β coefficients indicate an effect size of each independent variable on POR for CKD.

## Data Availability

All data are contained within this article.

## References

[1] Satarug S., Vesey D.A., Gobe G.C. (2017). Current health risk assessment practice for dietary cadmium: Data from different countries. Food Chem. Toxicol..

[2] Boon P.E., Pustjens A.M., Te Biesebeek J.D., Brust G.M.H., Castenmiller J.J.M. (2022). Dietary intake and risk assessment of elements for 1- and 2-year-old children in the Netherlands. Food Chem. Toxicol..

[3] Fechner C., Hackethal C., Höpfner T., Dietrich J., Bloch D., Lindtner O., Sarvan I. (2022). Results of the BfR MEAL Study: In Germany, mercury is mostly contained in fish and seafood while cadmium, lead, and nickel are present in a broad spectrum of foods. Food Chem. X.

[4] Aoshima K. (1987). Epidemiology of renal tubular dysfunction in the inhabitants of a cadmium-polluted area in the Jinzu River basin in Toyama Prefecture. Tohoku J. Exp. Med..

[5] Horiguchi H., Aoshima K., Oguma E., Sasaki S., Miyamoto K., Hosoi Y., Katoh T., Kayama F. (2010). Latest status of cadmium accumulation and its effects on kidneys, bone, and erythropoiesis in inhabitants of the formerly cadmium-polluted Jinzu River Basin in Toyama, Japan, after restoration of rice paddies. Int. Arch. Occup. Environ. Health.

[6] Kurata Y., Katsuta O., Doi T., Kawasuso T., Hiratsuka H., Tsuchitani M., Umemura T. (2014). Chronic cadmium treatment induces tubular nephropathy and osteomalacic osteopenia in ovariectomized cynomolgus monkeys. Vet. Pathol..

[7] Wong C., Roberts S.M., Saab I.N. (2022). Review of regulatory reference values and background levels for heavy metals in the human diet. Regul. Toxicol. Pharmacol..

[8] JECFA (2011). Summary and Conclusions. Proceedings of the Joint FAO/WHO Expert Committee on Food Additives and Contaminants, Seventy-Third Meeting.

[9] Satarug S., Vesey D.A., Nishijo M., Ruangyuttikarn W., Gobe G.C. (2019). The inverse association of glomerular function and urinary β2-MG excretion and its implications for cadmium health risk assessment. Environ. Res..

[10] Satarug S., Vesey D.A., Gobe G.C. (2022). Dose-response analysis of the tubular and glomerular effects of chronic exposure to environmental cadmium. Int. J. Environ. Res. Public Health.

[11] Satarug S., Vesey D.A., Ruangyuttikarn W., Nishijo M., Gobe G.C., Phelps K.R. (2019). The source and pathophysiologic significance of excreted cadmium. Toxics.

[12] Satarug S., Vesey D.A., Nishijo M., Ruangyuttikarn W., Gobe G.C., Phelps K.R. (2021). The effect of cadmium on GFR is clarified by normalization of excretion rates to creatinine clearance. Int. J. Mol. Sci..

[13] Schnaper H.W. (2017). The tubulointerstitial pathophysiology of progressive kidney disease. Adv. Chronic Kidney Dis..

[14] Sand S., Victorin K., Filipsson A.F. (2008). The current state of knowledge on the use of the benchmark dose concept in risk assessment. J. Appl. Toxicol..

[15] EFSA Scientific Committee (2017). Update: Use of the benchmark dose approach in risk assessment. EFSA J..

[16] Moffett D.B., Mumtaz M.M., Sullivan D.W., Whittaker M.H., Nordberg G., Costa M. (2022). Chapter 13, General Considerations of Dose-Effect and Dose-Response Relationships. Handbook on the Toxicology of Metals.

[17] Satarug S., Swaddiwudhipong W., Ruangyuttikarn W., Nishijo M., Ruiz P. (2013). Modeling cadmium exposures in low- and high-exposure areas in Thailand. Environ. Health Perspect..

[18] Yimthiang S., Pouyfung P., Khamphaya T., Kuraeiad S., Wongrith P., Vesey D.A., Gobe G.C., Satarug S. (2022). Effects of environmental exposure to cadmium and lead on the risks of diabetes and kidney dysfunction. Int. J. Environ. Res. Public Health.

[19] Denic A., Elsherbiny H., Rule A.D. (2019). In-vivo techniques for determining nephron number. Curr. Opin. Nephrol. Hypertens..

[20] Levey A.S., Becker C., Inker L.A. (2015). Glomerular filtration rate and albuminuria for detection and staging of acute and chronic kidney disease in adults: A systematic review. JAMA.

[21] Soveri I., Berg U.B., Björk J., Elinder C.G., Grubb A., Mejare I., Sterner G., Bäck S.E., SBU GFR Review Group (2014). Measuring GFR: A systematic review. Am. J. Kidney Dis..

[22] White C.A., Allen C.M., Akbari A., Collier C.P., Holland D.C., Day A.G., Knoll G.A. (2019). Comparison of the new and traditional CKD-EPI GFR estimation equations with urinary inulin clearance: A study of equation performance. Clin. Chim. Acta.

[23] Phelps K.R., Gosmanova E.O. (2020). A generic method for analysis of plasma concentrations. Clin. Nephrol..

[24] Slob W., Moerbeek M., Rauniomaa E., Piersma A.H. (2005). A statistical evaluation of toxicity study designs for the estimation of the benchmark dose in continuous endpoints. Toxicol. Sci..

[25] Slob W., Setzer R.W. (2014). 2014. Shape and steepness of toxicological dose-response relationships of continuous endpoints. Crit. Rev. Toxicol..

[26] Zhu Y., Wang T., Jelsovsky J.Z. (2007). Bootstrap estimation of benchmark doses and confidence limits with clustered quantal data. Risk Anal..

[27] Nichols G.A., Déruaz-Luyet A., Brodovicz K.G., Kimes T.M., Rosales A.G., Hauske S.J. (2020). Kidney disease progression and all-cause mortality across estimated glomerular filtration rate and albuminuria categories among patients with vs. without type 2 diabetes. BMC Nephrol..

[28] George C., Mogueo A., Okpechi I., Echouffo-Tcheugui J.B., Kengne A.P. (2017). Chronic kidney disease in low-income to middle-income countries: *Case Increased Screening*. BMJ Glob Health..

[29] George C., Echouffo-Tcheugui J.B., Jaar B.G., Okpechi I.G., Kengne A.P. (2022). The need for screening, early diagnosis, and prediction of chronic kidney disease in people with diabetes in low- and middle-income countries-a review of the current literature. BMC Med..

[30] Kalantar-Zadeh K., Jafar T.H., Nitsch D., Neuen B.L., Perkovic V. (2021). Chronic kidney disease. Lancet.

[31] Ferraro P.M., Costanzi S., Naticchia A., Sturniolo A., Gambaro G. (2010). Low level exposure to cadmium increases the risk of chronic kidney disease: Analysis of the NHANES 1999–2006. BMC Public Health.

[32] Navas-Acien A., Tellez-Plaza M., Guallar E., Muntner P., Silbergeld E., Jaar B., Weaver V. (2009). Blood cadmium and lead and chronic kidney disease in US adults: A joint analysis. Am. J. Epidemiol..

[33] Lin Y.S., Ho W.C., Caffrey J.L., Sonawane B. (2014). Low serum zinc is associated with elevated risk of cadmium nephrotoxicity. Environ. Res..

[34] Madrigal J.M., Ricardo A.C., Persky V., Turyk M. (2018). Associations between blood cadmium concentration and kidney function in the U.S. population: Impact of sex, diabetes and hypertension. Environ. Res..

[35] Tsai K.F., Hsu P.C., Lee C.T., Kung C.T., Chang Y.C., Fu L.M., Ou Y.C., Lan K.C., Yen T.H., Lee W.C. (2021). Association between enzyme-linked immunosorbent assay-measured kidney injury markers and urinary cadmium levels in chronic kidney disease. J. Clin. Med..

[36] Myong J.-P., Kim H.-R., Baker D., Choi B. (2012). Blood cadmium and moderate-to-severe glomerular dysfunction in Korean adults: Analysis of KNHANES 2005–2008 data. Int. Arch. Occup. Environ. Health.

[37] Chung S., Chung J.H., Kim S.J., Koh E.S., Yoon H.E., Park C.W., Chang Y.S., Shin S.J. (2014). Blood lead and cadmium levels and renal function in Korean adults. Clin. Exp. Nephrol..

[38] Park Y., Lee S.J. (2022). Association of blood heavy metal levels and renal function in Korean adults. Int. J. Environ. Res. Public Health.

[39] Jalili C., Kazemi M., Cheng H., Mohammadi H., Babaei A., Taheri E., Moradi S. (2021). Associations between exposure to heavy metals and the risk of chronic kidney disease: A systematic review and meta-analysis. Crit. Rev. Toxicol..

[40] Byber K., Lison D., Verougstraete V., Dressel H., Hotz P. (2016). Cadmium or cadmium compounds and chronic kidney disease in workers and the general population: A systematic review. Crit. Rev. Toxicol..

[41] Liu C., Li Y., Zhu C., Dong Z., Zhang K., Zhao Y., Xu Y. (2016). Benchmark dose for cadmium exposure and elevated N-acetyl-β-D-glucosaminidase: A meta-analysis. Environ. Sci. Pollut. Res. Int..

[42] Pócsi I., Dockrell M.E., Price R.G. (2022). Nephrotoxic biomarkers with specific indications for metallic pollutants: Implications for environmental health. Biomark. Insights.

[43] Suwazono Y., Sand S., Vahter M., Filipsson A.F., Skerfving S., Lidfeldt J., Akesson A. (2006). Benchmark dose for cadmium-induced renal effects in humans. Environ. Health Perspect..

[44] Qing Y., Yang J., Zhu Y., Li Y., Zheng W., Wu M., He G. (2021). Dose-response evaluation of urinary cadmium and kidney injury biomarkers in Chinese residents and dietary limit standards. Environ. Health.

[45] Shi Z., Taylor A.W., Riley M., Byles J., Liu J., Noakes M. (2018). Association between dietary patterns, cadmium intake and chronic kidney disease among adults. Clin. Nutr..

[46] EFSA (2011). European Food Safety Agency, Statement on tolerable weekly intake for cadmium. EFSA J..

[47] Repić A., Bulat P., Antonijević B., Antunović M., Džudović J., Buha A., Bulat Z. (2020). The influence of smoking habits on cadmium and lead blood levels in the Serbian adult people. Environ. Sci. Pollut. Res. Int..

[48] Pappas R.S., Fresquez M.R., Watson C.H. (2015). Cigarette smoke cadmium breakthrough from traditional filters: Implications for exposure. J. Anal. Toxicol..

[49] Ferraro P.M., Sturniolo A., Naticchia A., D’Alonzo S., Gambaro G. (2012). Temporal trend of cadmium exposure in the United States population suggests gender specificities. Intern. Med. J..

[50] Tellez-Plaza M., Navas-Acien A., Caldwell K.L., Menke A., Muntner P., Guallar E. (2012). Reduction in cadmium exposure in the United States population, 1988–2008: The contribution of declining smoking rates. Environ. Health Perspect..

[51] Callan A., Hinwood A., Devine A. (2014). Metals in commonly eaten groceries in Western Australia: A market basket survey and dietary assessment. Food Addit. Contam. A.

[52] Arnich N., Sirot V., Riviere G., Jean J., Noel L., Guerin T., Leblanc J.-C. (2012). Dietary exposure to trace elements and health risk assessment in the 2nd French Total Diet Study. Food Chem. Toxicol..

[53] González N., Calderón J., Rúbies A., Timoner I., Castell V., Domingo J.L., Nadal M. (2019). Dietary intake of arsenic, cadmium, mercury and lead by the population of Catalonia, Spain: Analysis of the temporal trend. Food Chem. Toxicol..

[54] Butler-Dawson J., James K.A., Krisher L., Jaramillo D., Dally M., Neumann N., Pilloni D., Cruz A., Asensio C., Johnson R.J. (2022). Environmental metal exposures and kidney function of Guatemalan sugarcane workers. J. Expo. Sci. Environ. Epidemiol..

[55] Win-Thu M., Myint-Thein O., Win-Shwe T.-T., Mar O. (2021). Environmental cadmium exposure induces kidney tubular and glomerular dysfunction in the Myanmar adults. J. Toxicol. Sci..

[56] Skröder H., Hawkesworth S., Kippler M., El Arifeen S., Wagatsuma Y., Moore S.E., Vahter M. (2015). Kidney function and blood pressure in preschool-aged children exposed to cadmium and arsenic-potential alleviation by selenium. Environ. Res..

[57] Rodríguez-López E., Tamayo-Ortiz M., Ariza A.C., Ortiz-Panozo E., Deierlein A.L., Pantic I., Tolentino M.C., Estrada-Gutiérrez G., Parra-Hernández S., Espejel-Núñez A. (2020). Early-life dietary cadmium exposure and kidney function in 9-year-old children from the PROGRESS cohort. Toxics.

